# Impact of Airborne Particle Morphology on Filtration Processes

**DOI:** 10.3390/ma18163781

**Published:** 2025-08-12

**Authors:** Franco Furgiuele, Lucija Boskovic, Igor E. Agranovski

**Affiliations:** 1 School of Engineering and Built Environment, Griffith University, Brisbane, QLD 4111, Australia; franco.furgiuele@griffithuni.edu.au; 2 Business and Hospitality Faculty, Torrens University, 90 Bowen Tce, Brisbane, QLD 4006, Australia; lucija.boskovic@torrens.edu.au

**Keywords:** airborne particle morphology, gas filtration, filter efficiency, air pollution

## Abstract

This study explores the critical role of airborne nanoparticle shape in air filtration performance, with direct relevance to the field of nanomaterials production. Aerosol particles ranging from 40 to 250 nm—including spherical Fe_2_O_3_, cubic MgO, straight rod-shaped ZnO, and curved or clustered COOH-functionalized nanotubes—were synthesized and tested to assess shape-dependent filtration behavior. The results indicate that the effect of particle morphology on filtration efficiency becomes markedly pronounced at larger particle sizes. For instance, at 250 nm, filtration efficiency differed by as much as 30% between spherical Fe_2_O_3_ and rod-shaped ZnO particles. These findings have substantial implications for industries engaged in large-scale nanomaterial synthesis, particularly where anisotropic or rod-like particles are prevalent. The potential for higher-than-anticipated atmospheric release of such particles underscores the need for refined environmental controls and monitoring. Furthermore, the current practice of using primarily spherical particles in air filter certification tests may require reconsideration to ensure accuracy and applicability to real-world scenarios involving non-spherical nanomaterials.

## 1. Introduction

Filtration stands as the most traditional and widely utilized method for managing aerosols within gas streams. Classical filtration theory, developed primarily in the latter half of the 20th century, focused on particle-capturing mechanisms such as interception, diffusion, and inertial impaction, typically assuming particles to be homogeneous spheres to simplify theoretical modeling [1,2]. As a result, the influences of particle morphology, such as aspect ratio, surface roughness, and shape irregularity, on filtration efficiency were often overlooked or only minimally addressed. Although the most recent edition of [3] acknowledges that airborne particles frequently deviate from idealized spherical forms, it offers limited quantitative treatment of how these morphological complexities affect filtration performance in fibrous media. Such oversight can lead to measurable discrepancies between theoretical and real-world filtration efficiencies, particularly in fibrous filters operating under practical conditions where particle bounce and re-entrainment occur due to non-spherical shapes and surface interactions [4,5]. This discrepancy is particularly relevant to industries that generate irregular or anisotropic aerosols, such as metallurgy, ceramic manufacturing, and fiber processing, where the assumption of spherical particle geometry may significantly underestimate filtration behavior and device performance. It is now well recognized that particles produced or used in many industrial and engineered applications exhibit a wide range of shapes, yet continue to be modeled predominantly as spheres, despite growing evidence that particle morphology significantly influences aerodynamic behavior, surface interactions, and material performance across scales [6]. Moreover, recent computational studies have reinforced the importance of particle morphology; for example, [7] demonstrated through CFD-DEM simulations that non-spherical particles, such as triangular shapes, deposit differently on filter fibers compared to spheres of the same volume, substantially affecting capture efficiency. With the increasing prevalence of engineered nanomaterials with complex geometries, modern studies have begun to more rigorously examine the role of particle shape in filtration efficiency and capture dynamics, underscoring the need to revise or expand traditional filtration models accordingly.

It is noteworthy that a significant body of literature delves into the dynamic behavior of non-spherical particles. Hinds [2], for example, provided a comprehensive overview by introducing the Dynamic Shape Factor (DSF), which compares dynamics of non-spherical particles to perfect spheres. Various shapes, including cubes, cylinders, and chains, were examined, and DSF values were derived accordingly and can be generalized to all types.

Also, very interesting results were reported by Gradon and colleagues [8], who investigated large aerosol particles with diameters of up to 10 µm. They applied classical single-fiber filtration theory to elongated (fibrous) aerosol particles by incorporating their shape and rotation into models, rather than treating them as spheres. It was found that, compared to spherical particles of equal mass, elongated particles exhibit lower absolute deposition efficiency due to their streamlined shape, but their overall efficiency increases when accounting for orientation effects (configurational efficiency).

With regard to the airborne nanoparticles, previous research examined the influence of their size and shape on the efficiency of filtering materials in chemical protective clothing (CPC) and demonstrated that while particle size had minimal impact on penetration levels due to diffusion-based collection mechanisms at low face velocity, nanoparticle shape played a significant role [9]. The study assessed the effect of size using 30 and 300 nm nSiO_2_ and the effect of morphology using 30–50 nm anatase and rutile nTiO_2_. The findings revealed that spherical particles (nTiO_2_ anatase) exhibited lower penetration compared to rod-shaped ones (nTiO_2_ rutile), underscoring the importance of shape in filtration efficiency. Another study investigated how the size and morphology of 25 nm TiO_2_ nanoparticles affect filtration through a screen filter with a packing density of 0.345 and face velocities ranging from 2.92 to 5.85 cm/s [10]. Their analysis revealed that agglomerates formed during aerosol generation exhibited reduced penetration compared to individual particles.

Several studies were undertaken on filtration of different types of microbial and mineral ultrafine aerosols. For example, the performance of filtering facepiece respirators (N99 and N95) was challenged with an inert aerosol (NaCl) and three viral aerosols (enterobacteriophages MS2 and T4 and bacteriophage SP01), with respirators placed and sealed on manikins [11]. The results showed no significant differences, suggesting that inert NaCl aerosols may generally be appropriate for modeling filter penetration of similarly sized virus particles.

Filtration of non-spherical nanomaterials, such as carbon nanotubes and nanoparticle agglomerates, has been investigated, and it was demonstrated that carbon nanotubes, owing to their non-spherical shape, exhibit distinct filtration behavior. Their elongated shape enhances interception effects, resulting in superior filtration efficiency compared to spherical particles [12]. This study employed modeling based on the single-fiber theory to predict variations in filter penetration between agglomerates, carbon nanotubes, and spheres. Experimental findings suggest that carbon nanotubes and nanoparticle agglomerates exhibit greater filtration efficiency compared to spheres with equivalent mobility. Analysis of the filtration model suggests that the enhanced interception effect primarily accounts for this disparity.

The toxicological effects of nanomaterials and their underlying mechanisms of action remain an area of intense global investigation, driven by both scientific interest and regulatory necessity. Among these mechanisms, particle morphology, specifically shape, emerges as a critical determinant of cytotoxicity [13]. For example, [14] reported that the shape of nano-hydroxyapatite particles significantly influences their genotoxic potential, with round forms inducing greater DNA damage than rod-shaped ones, highlighting the critical role of nanoparticle morphology in toxicity mechanisms. Additionally, contemporary reviews reinforce the observation that non-spherical metal-based nanoparticle architectures display distinct biodistribution and cellular uptake behaviors compared to spherical counterparts, even when size and composition are held constant [15,16]. These findings underscore the urgent need to integrate morphological parameters into nanotoxicological risk assessments, ensuring safer design and use of engineered nanomaterials in biomedical, industrial, and consumer settings.

In the realm of targeted drug delivery, the size and morphology of drug nanocarriers hold significant importance, prompting investigation into various techniques for detecting nanocarriers during cellular uptake. Building on a comprehensive review of current studies, it has been suggested that nanocarriers possessing a rod-shaped morphology and a narrow cross-section exhibit an enhanced capability in penetrating cell membranes compared to their counterparts of differing shapes [17]. Furthermore, investigations have highlighted the substantial influence of the orientation of non-spherical nanocarriers, such as nanorods, on the cellular uptake process.

For perfectly spherical particles, as demonstrated by Boskovic et al. [5], the particle that comes into contact with the fiber either rolls or slides. However, the scenario would be entirely different in the case of non-spherically shaped particles (such as cubes), which either slide on a face or tumble on a corner or edge. Obviously, the particle size (and correspondingly the surface area) also plays a crucial role in the bouncing processes.

Particle shape analysis presents a significant challenge in powder technology due to the diverse and intricate geometries of particles, which influence their behavior in various processes such as filtration, flow, and packing. Accurately characterizing particle shape is essential for understanding and predicting these behaviors [18]. In the context of electrostatic classifiers, the relationship between electrical mobility and particle shape is particularly important. Electrical mobility is influenced not only by particle size but also by its shape and charge distribution. While spherical particles exhibit a relatively straightforward relationship between size and electrical mobility, non-spherical particles display more complex behaviors due to factors such as surface area, aspect ratio, and their orientation within the electric field [2]. For example, non-spherical particles can experience higher or lower electrical mobility compared to spherical ones of the same volume, depending on factors like their surface area and how the charge is distributed across the particle. The orientation of non-spherical particles within the electric field can also affect their interaction with the field, influencing their overall mobility.

This study experimentally investigates the removal efficiency of nanosized particles with various shapes on a moderate-efficiency fibrous filter. The use of a moderate-efficiency filter provides a more comprehensive and accurate understanding of how particle shape affects filtration efficiency. The experimental results are then compared to the theoretical filtration efficiency, which is calculated using the widely accepted total single-fiber efficiency concept [2].

We would like to note a very important development that has allowed us to produce this concise yet crucial addition to our previous work published approximately 20 years ago [5]. At that time, our research was limited to nanoparticles of two ideal geometric shapes: cubic MgO and spherical PSL/Fe_2_O_3_. These enabled us to draw important conclusions regarding their behavior during filtration processes. Despite our extensive efforts to find a “single dimension” nanoobject that could demonstrate unpredictable filtration results, we were unsuccessful, as most fibrous aerosols were well outside the nanoscale range. The current manuscript utilizes rod-shaped ZnO nanoparticles, which we have just recently managed to synthesize. Additionally, short nanotubes (up to 1 µm), which were previously unavailable, are now commercially accessible, allowing us to investigate their behavior during their removal from the air carrier by fibrous filters. Furthermore, the two shapes we previously studied (cubes and spheres) were reinvestigated in this project to facilitate a direct comparison of the results obtained for nanoobjects with different geometries.

## 2. Experimental

In this research four different types of nanoparticles were used, including spherical iron oxide nanoparticles (Fe_2_O_3_, density—5.2 g/cm^3^), cubic magnesium oxide nanoparticles (MgO, density—3.6 g/cm^3^), straight rod-shaped zinc oxide (ZnO, density—5.61 g/cm^3^) and curved nanotubes COOH (functionalized multi-walled carbon nanotubes with a density ~1.9 g/cm^3^, obtained from Cheap Tubes Inc., Grafton, VT, USA). The direct metal combustion procedures, outlined in detail in our previous works [5,19], were used to produce all three metal nano-oxides mentioned above. In brief, pure metals were subjected to combustion, resulting in the formation of nanoparticles in the gas phase. These nanoparticles were collected on a suitable substrate, carefully brushed from the surface, and added to nano-pure deionized water with a resistivity of >18.0 MΩ to produce highly concentrated suspensions. The suspensions underwent sonication for a minimum of 15 min to minimize the number of agglomerates potentially present in the nano-powder after collection on the substrate.

Short functionalized multi-walled carbon nanotubes COOH were utilized directly in the experiments. Similarly to the metal nano-powders, the nanotubes were added to the nano-pure deionized water and subjected to sonication for the same duration as the other suspensions.

**Figure 1:**
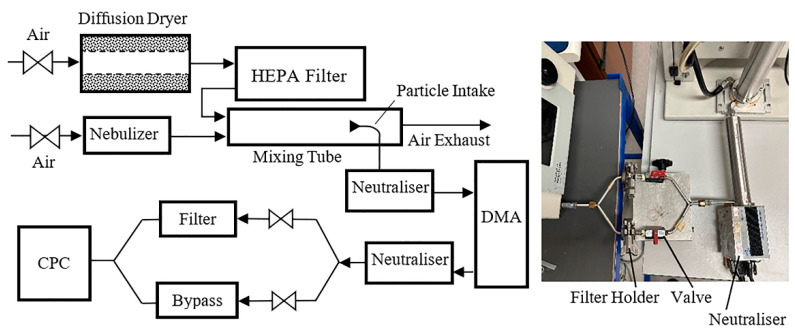
Laboratory setup (inset illustrates arrangement of the neutralizer in the aerosol line).

Figure 1 shows the experimental arrangement employed in this study, which was similar to the one previously described by Boskovic et al. [5]. A portion of the concentrated particle suspension (either iron oxide, magnesium oxide, zinc oxide, or nanotubes), prepared as described earlier, was gradually introduced, in 0.1 mL increments, into approximately 20 mL of nano-pure water within a single jet Collison nebulizer. This process ensured the generation of a representative quantity of particles for each size employed in the experiments. Subsequently, the aerosolization was accomplished using a flow of 2 L/min of compressed ultra-high purity air to produce the test aerosol. To ensure effective drying of moisture from the particle surface, the aerosol stream was combined in a mixing tube with 10 L/min of dry HEPA-filtered air and passed through a charge neutralizer (10-mCi85Kr, model 3012, TSI Inc., Shoreview, MN, USA) before reaching an Electrostatic Classifier (EC) (Model 3080N, TSI Inc., Shoreview, MN, USA) containing a DMA column (Model 3081, TSI Inc., Shoreview, MN, USA). This charge neutralizer imparts a predictable charge distribution to aerosol particles, effectively minimizing the impact of electrostatic forces and ensuring that particle charges do not interfere with the subsequent filtration measurements. To ensure equilibrated charge on all particles involved in the experiments, the neutralizer (Model 3088, TSI Inc., Shoreview, MN, USA) was also used after the DMA column at the filter entry point for all types of particles in all experimental runs (see inset in Figure 1).

To ensure high accuracy and integrity in the results, instead of using standard piping typically employed to connect the DMA column and the Condensation Particle Counter (CPC) (Model 3775, TSI Inc., Shoreview, MN, USA), a custom-made connector previously described by Boskovic et al. [5] was utilized in the experiments (Figure 1). The connector was composed of two identical branches consisting of 21 mm diameter filter holders and valves having a mutual air inlet and outlet. During the experiments, a filter medium was placed randomly in one of the filter holders, leaving the second line empty. During the experiment, one of the lines was closed by the corresponding valve, forcing the aerosol to pass through the second one. On completion of the measurement, the valves were altered, exposing the second line for the aerosol flow. The number of particles counted by the Condensation Particle Counter (CPC) after passing through the filter represented the downstream concentration, while particles counted after the bypass line corresponded to the initial (upstream) concentration of aerosols. Such a configuration proved advantageous in obtaining results for the calculation of the filter efficiency without the need for frequent assembly or disassembly of filter holders and altering of sampling points, which might be associated with some inaccuracies in the results.

A minimum of five experimental repeats were conducted for each particle size to ensure reliable and reproducible results. Throughout the experiments, all conditions (e.g., airflow, particle size distribution, and environmental factors) were carefully controlled and kept constant. Flow rates were continuously monitored and regulated using flowmeters (Model 5200, TSI Inc., Shoreview, MN, USA) and adjustable valves. The experimental filtration efficiency results were then compared with the theoretical filtration efficiency E, commonly defined in the literature as the following [20]:

(1) $$E=1-exp\left(\frac{-4\alpha E_{\sum }t}{\pi (1-{\alpha )d}_{f}}\right)$$

where *α* is the filter packing density or solidity, *t* is the filter thickness, *d_f_* is the fiber diameter, and *E_∑_* is the total single-fiber efficiency, which is typically determined as the sum of all filtration mechanisms [2]:

(2) $$E_{\sum }\approx E_{R}+E_{I}+E_{D}+E_{DR}$$

where $E_{R}$ is the single fiber efficiency for interception, $E_{I}$ is the single fiber efficiency for impaction, $E_{D}$ is the single fiber efficiency due to diffusion, and to account for the enhanced collection due to interception of the defusing particles, it is also necessary to include an interaction term, $E_{DR}$. All these mechanisms are commonly calculated according to the equations provided by Hinds [2]. Electrostatic attraction was excluded from consideration, as the majority of particles exiting the DMA column carry a unit charge, resulting in similar behavior regardless of particle shape. The impact of fiber charging was also deemed negligible, as the experimental conditions were consistent for all particle types. As noted previously, the charge neutralizer imparts a predictable charge distribution to aerosol particles, minimizing electrostatic effects and ensuring that particle charges do not interfere with subsequent filtration measurements. Additionally, according to [2], particle mobility under typical charging conditions is a relatively weak function of particle size in the range of 0.1 to 1 µm, suggesting that electrostatic effects have limited influence in this size range. Given that the particles in this study fall within or near this range, electrostatic mechanisms can be reasonably excluded from the filtration efficiency calculations, allowing for a more accurate assessment of the physical filtration mechanisms.

Various researchers have proposed mathematical expressions for efficiency attributed to individual filtration mechanisms, both through analytical and empirical approaches. A comprehensive review of these expressions can be found in [2]. It is often observed that a singular mechanism tends to prevail, leading to the assumption that this mechanism solely governs the overall filtration efficiency. Both interception and impaction prove negligible for small particles but exhibit a rapid increase for particles larger than 300 nm. Conversely, diffusion emerges as the predominant mechanism for particles below 200 nm. With these competing filtration mechanisms being most effective across different size ranges, all filters have a particle size that provides minimum efficiency for a specific set of process parameters, defined as the most penetrating particle size (MPPS). As broadly reported in the literature, the MPPS can have quite a wide range of magnitude; however, it is usually found within a range between 50 and 350 nm (see, for ex., [21]).

**Figure 2:**
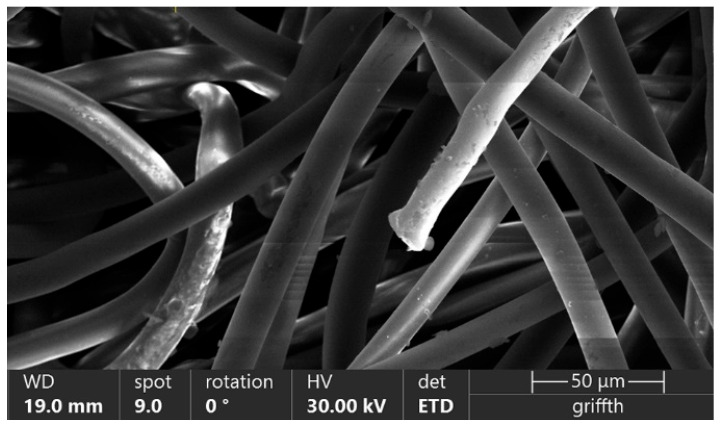
Fibrous filter used in experiments.

A fresh, 2.0 mm thick, polypropylene filter with a packing density of 8% and a fiber diameter of 15 μm was used in all the experiments (Figure 2). It should be noted that using such a moderate-efficiency filter provides a more thorough and accurate picture of how the particle shape affects filtration efficiency. It ought to be noted that, in contrast, using low-efficiency filters would be associated with almost zero values obtained for the sizes around the MPPS, which would significantly mask any effects related to the subject of this investigation. Obviously, the effect would be the opposite in cases using highly efficient filter media; the results would be close to 100% for more easily collectable particles representing the low-size end used in this project, significantly complicating any analysis of the outcomes. In addition, the results of the research will be compared to our previously reported results for cubic and spherical particles obtained for fibrous filters with different geometries. Such comparisons are very important for the generalization of conclusions to be drawn from this study.

## 3. Results and Discussion

**Figure 3:**
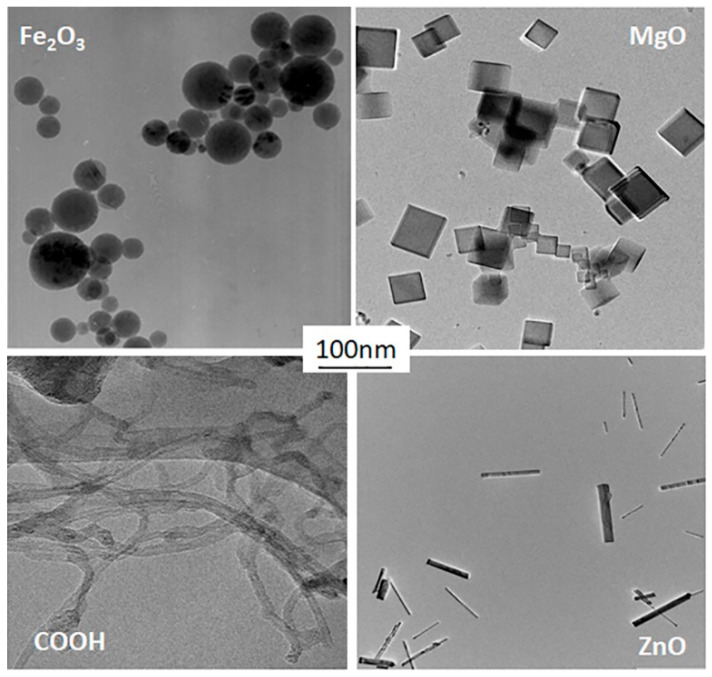
Experimental particles (spherical Fe_2_O_3_, cubic MgO, curved fibers of COOH, and straight rods of ZnO).

Figure 3 shows a typical TEM image of iron oxide (Fe_2_O_3_), magnesium oxide (MgO), curved nanotube (COOH), and zinc oxide (ZnO) particles collected from the nebulizer suspension on a TEM grid and visualized by Transmission Electron Microscope (Model 2100, JEOL, Tokyo, Japan). Figure 3 clearly demonstrates that the collected nanoparticles are non-agglomerated in a wide range of sizes. A very important fact is that all types of metal oxide particles used in this project have perfect geometry (sphere, cube, and straight rod), enabling us to draw clear conclusions on the behavior of different shapes in the filtration process. The COOH nanoparticles are also crucial for this investigation, as their curvature is a very interesting parameter for comparison against straight nanorods of zinc oxide.

**Figure 4:**
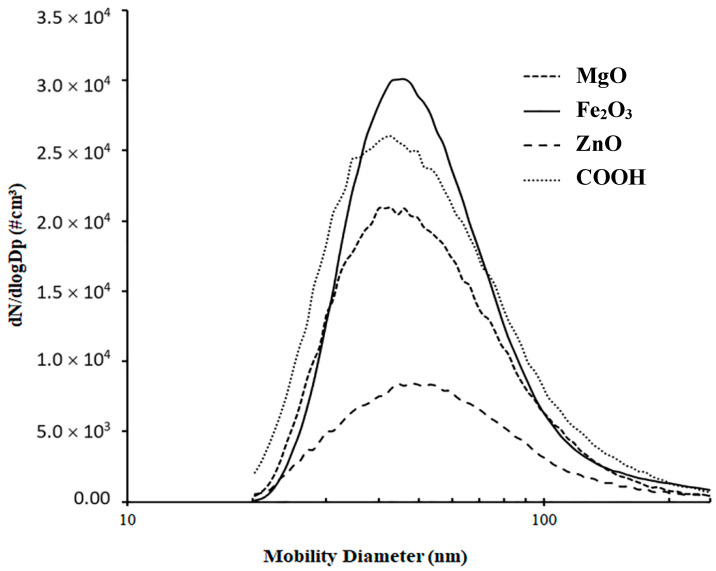
Particle size distribution at the tested filter inlet.

Before experiments with each type of particle, the size distribution was obtained to ensure that a representative number of at least 1000 cm^−3^ particles for each size used in the experiments could be generated. As discussed before, to obtain these values, small aliquots of the concentrated suspension, prepared as described above, were gradually added to 20 mL of nano-pure water in the nebulizer, and the device was operated. The filtration flow rate of 0.3 L/min (based on the operation flow rate of the CPC), which was associated with the filter face velocity of 14 mm/s, was used in all the experiments. The size distribution scan, obtained by the EC operating in the “Analog control” mode, was adjusted by adding more aliquots of the concentrated particle suspension until the required concentrations were achieved. This step is very important, as excessive numbers of particles used in the experiments could exceed the upper limit for reliable instrument operation (5.5 × 10^4^ cm^−1^) and introduce some errors to the experimental results. Figure 4 illustrates the results of the optimized size distribution obtained from the “bypass” line for all types of particles. As is seen, all the tested aerosols exhibit reasonably similar trends with sufficient numbers of particles obtained for all sizes within the range of 40–250 nm.

The next step involved switching the EC to the “Panel control” mode, setting the sampling rate to 0.3 lpm and the sheath air flow rate to 3 lpm, and selecting the first size of 40 nm on the instrument panel. The size distribution and concentration data were collected from both lines over a 2 min period each, altering the lines 10 times (five runs for each line). Then, the next particle size of 70 nm was selected, and the procedure was repeated. The same protocol was used for all the remaining sizes. On completion, a fresh filter was placed into the holder, the nebulizer was charged with a fresh suspension, and the procedure was repeated twice more. Then three identical repeats were undertaken for all the remaining particle types used in this study. Finally, the experimental filtration efficiency was calculated according to the following classic equation [1]:

(3) $$E_{T}=\left(1-\frac{C_{B}}{C_{A}}\right)\times 100\%$$

**Figure 5:**
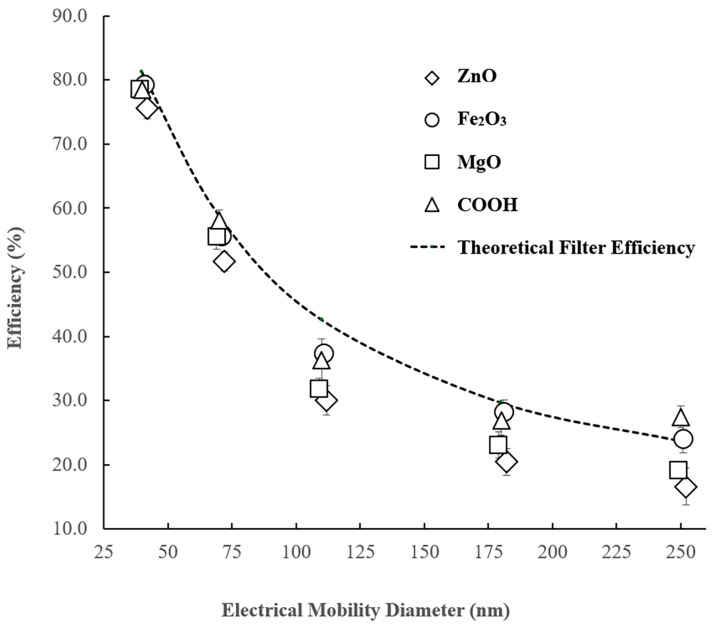
Filter efficiency for removal of airborne particles of different shapes (dashed line shows the theoretical removal efficiency calculated by the classic filtration theory).

The concentrations of particles passing the bypass line (upstream concentration) and filter line (downstream concentration) are denoted by *C_A_* and *C_B_*, respectively. Figure 5 depicts the experimental filter efficiency for all the investigated particles, alongside the theoretical filter efficiency estimated by Equations (1) and (2) for the filter and process parameters outlined above. The error bars, representing the standard deviation of all five experimental runs, are included. The main outcomes derived from Figure 5 can be summarized as follows:

- It must be noted that the theoretical efficiency is estimated based on the particle’s geometric diameter, whilst the EC separates the sizes based on their electrical mobility. These numbers can only perfectly coincide for spherical particles, so, generally speaking, the theoretical results can only be precisely compared against experimental values obtained for the spherical particles. However, in this study, the theoretical curve primarily serves as a “scale factor” for reference, with the primary focus placed on comparisons of the experimental results obtained for different particle types. Notably, the alignment between the theoretical and experimental efficiency curves for spherical Fe_2_O_3_ nanoparticles is exceptionally close. This agreement strongly suggests that electrostatic effects, including those from residual particle charges or filter charging, were minimal and did not significantly influence filtration efficiency. While multiple charging is possible for particles around 300 nm, the previous literature (e.g., [2]) indicates that the fraction of multiply charged particles at this size is relatively modest, with approximately 9% of particles being double charged. Any potential misclassification due to multiple charging would likely have introduced discrepancies between the theoretical and experimental efficiency curves, particularly for particles near 250 nm. The absence of such discrepancies confirms that multiple charging effects were not significant in this study.
- In light of these factors, we conclude that the pre-DMA neutralization effectively controlled for electrostatic effects, making the consideration of using a second neutralizer between the DMA and the filter holder unnecessary for isolating the physical filtration mechanisms examined in this study. The experimental results for particle sizes ranging from 40 to 250 nm consistently exhibited an anticipated trend: the efficiency decreased towards the MPPS, which theoretically is supposed to be between 200 and 300 nm. This observed trend is well expected and common for all gas filtration processes.
- Spherical particles exhibit higher removal efficiency compared to the investigated cubic and straight rod particles. For example, the filtration efficiency of spherical iron oxide nanoparticles with a size of 250 nm was 24.2%, while the removal efficiency of cubic and straight rod particles with the same mobility size was approximately 19.1% and 16.6%, respectively. This result means that, even for relatively narrow ranges of the process parameters used in this study, the difference in filtration efficiency can exceed 31% for different particle types and certain sets of parameters.
- The lowest efficiency was observed in the removal of short ZnO nanorods, which could be attributed to their smallest projection size when passing through the filter pores perpendicular to the filter face. This effect is expected to become more pronounced with an increase in particle size, as the ratio of particle size to filter pore size increases. Additionally, an increase in particle size is associated with an increase in the Reynolds number, leading to a corresponding enhancement in the alignment efficiency of fibers with the airflow streamlines [2], resulting in a greater number of rods with smaller projection sizes relative to the filter face.
- Nanotubes demonstrated either similar or slightly higher efficiency compared to the spherical particles, a characteristic that can be attributed to their larger outline and projection size. This unique morphology makes it easier for nanotubes to adapt to the curvature of the fibers and pore shapes and become more efficiently entrapped within the filter matrix. This outcome is in excellent agreement with the results reported by Wang [12].
- Intermediate efficiency was observed for MgO nano-cubes, aligning well with the findings presented in [22], where such an outcome was explained by bouncing of particles with a cubic shape after colliding with the fibers.
- The results of the current study for cubic and spherical particles are in excellent agreement with those obtained for filters with different geometries, as demonstrated in [5,22]. This consistency supports the conclusion that the findings of this study can be generalized to a broad range of processes employing fibrous filters for the removal of elongated nanoparticles in air quality control applications.

## 4. Concluding Remarks

This research underscores the critical role of particle morphology in filtration processes, providing valuable insights into the behavior of various particle shapes during filtration. Based on the analysis of the experimental results focusing on particles of different shapes, several key observations can be made:

1. The filtration efficiency of cubic particles and straight rods is lower compared to that of spherical and curved nanotube particles across most investigated particle sizes. This disparity highlights the significant influence of particle shape on filtration efficiency, which can be attributed to differences in particle motion and orientation during the filtration process.
2. The differences in filtration efficiency among larger particles are considerably more pronounced than those observed for smaller particles within the tested diameter range. This finding emphasizes the need to account for particle size and shape when evaluating filtration performance.
3. Perhaps the most significant outcome of this research is the realization that spherical aerosol particles, commonly used in air filter testing, may not represent the most penetrable particle shapes. This insight suggests a need to reconsider the selection of aerosol types for filter performance characterization, potentially adopting particle shapes that better reflect real-world filtration challenges.

## Data Availability

The original contributions presented in the study are included in the article, further inquiries can be directed to the corresponding author.
